# Effect of secular trends on age-related trajectories of cardiovascular risk factors: the Whitehall II longitudinal study 1985–2009

**DOI:** 10.1093/ije/dyt279

**Published:** 2014-01-24

**Authors:** Adam Hulmán, Adam G Tabák, Tibor A Nyári, Dorte Vistisen, Mika Kivimäki, Eric J Brunner, Daniel R Witte

**Affiliations:** ^1^Department of Medical Physics and Medical Informatics, University of Szeged, Szeged, Hungary, ^2^Department of Epidemiology and Public Health, University College London, London, UK, ^3^First Department of Medicine, Semmelweis University Faculty of Medicine, Budapest, Hungary, ^4^Steno Diabetes Center, Gentofte, Denmark and ^5^Centre de Recherche Public de la Santé, Strassen, Luxembourg

**Keywords:** Obesity, blood pressure, cholesterol, secular trend, ageing, quantile regression

## Abstract

**Background:** Secular trends in cardiovascular risk factors have been described, but few studies have examined simultaneously the effects of both ageing and secular trends within the same cohort.

**Methods:** Development of cardiovascular risk factors over the past three decades was analysed using serial measurements from 10 308 participants aged from 35 to 80 years over 25 years of follow-up from five clinical examination phases of the Whitehall II study. Changes of body mass index, waist circumference, blood pressure and total and high-density lipoprotein cholesterol distribution characteristics were analysed with quantile regression models in the 57–61 age group. Age-related trajectories of risk factors were assessed by fitting mixed-effects models with adjustment for year of birth to reveal secular trends.

**Results:** Average body mass index and waist circumference increased faster with age in women than in men, but the unfavourable secular trend was more marked in men. Distributions showed a fattening of the right tail in each consecutive phase, meaning a stronger increase in higher percentiles. Despite the higher obesity levels in younger birth cohorts, total cholesterol decreased markedly in the 57–61 age group along the entire distribution rather than in higher extremes only.

**Conclusion:** The past three decades brought strong and heterogeneous changes in cardiovascular risk factor distributions. Secular trends appear to modify age-related trajectories of cardiovascular risk factors, which may be a source of bias in longitudinal analyses.

**Key Messages**

- In recent years more people have high body mass index and waist circumference and fewer people have average values, but the proportion of people with low values has not changed.
- Whereas both systolic and diastolic blood pressure levels have declined in the past decades, diastolic blood pressure has done so more markedly, leading to an increasing pulse pressure.
- In the past decades total cholesterol levels have dropped in the entire population.
- Descriptions of risk factor trends focusing only on mean levels lack detail with regard to changing distributions.
- Secular trends should be taken into account when analysing age-related trajectories of risk factors to avoid biased estimates caused by large differences between successive birth cohorts.

## Introduction

Cardiovascular disease (CVD) remains the most common cause of death in the UK. Despite the fact that CVD mortality in the UK has halved during the past three decades, it still accounts for approximately 30% of total mortality.^1^ A very similar situation is seen on a global scale,^2^ mostly driven by the combination of ageing populations, sedentary lifestyle and high-calorie diets. Many CVD risk factors are increasingly controlled or treated (e.g. smoking, obesity, hypertension, diabetes, hypercholesterolaemia) in people at the high end of the risk distribution, and in many countries population-based campaigns have been put in place to promote a healthier lifestyle.

The combined effects of societal trends that increase CVD risk factors and the individual and population-based efforts to mitigate them have been different for different risk factors. Although systolic blood pressure and cholesterol levels have decreased markedly in high-income countries in the past decades,^3–9^ the obesity ‘epidemic’ seems to continue unabated.^10–17^

Much of the evidence on the trends in cardiovascular risk comes from the comparison of observed average risk factor levels in sequential cross-sectional surveys, and only few of these studies have investigated trends in the most important CVD risk factors within the same population.^7^^,^^8^

A second aspect that has received little attention is the change in cardiovascular risk factor distributions. This issue may have important practical implications because the Rose prevention paradigm, which has strongly affected public health policy in the past decades, assumes that as populations move into higher CVD risk levels, risk factor distributions shift in their entirety to the right. Hence, prevention of CVD events should target the entire population.^18^^,^^19^ However, few studies have examined whether the shift of CVD risk factor distributions indeed follows these assumptions. The limited evidence available for BMI suggests that the distribution has become increasingly skewed in the past decades, with little upward shift of the entire curve.^20–22^

To address these issues, we investigated the development of body mass index (BMI), waist circumference (WC), systolic and diastolic blood pressure (SBP and DBP), and total and high-density lipoprotein cholesterol (TC and HDL) over 25 years in a British occupational cohort, the Whitehall II study. Our aim was to determine whether (i) changes over time were similar along the full range of risk factor distributions, and whether (ii) age-related trajectories were affected by secular trends.

## Methods

### Study setting

Between 1985 and 1988, 10 308 British men and women (73% of those invited), aged 35–55 years and employed in London-based government departments, participated in the first phase of the Whitehall II study.^23^ Two thirds of them were men. Clinical examinations in addition to postal questionnaires were part of every second phase, i.e. phase 1 in 1985–88, phase 3 in 1991–94, phase 5 in 1997–99, phase 7 in 2002–04 and phase 9 in 2007–09. The final dataset contained up to five repeated measurements per participant.

### Measurements

BMI was calculated as the ratio of weight (in kg) and height-squared (in m^2^). WC (smallest) was first measured at phase 3. All anthropometric measures were assessed by trained nurses according to standardized protocols. Systolic and diastolic blood pressures were measured twice at each clinical phase and the average was used in the analyses. After phase 5, the manual random zero sphygmomanometer (MRZ) was switched to an automated oscillometric device (AOD). The biochemical analysis of blood samples to assess total and HDL cholesterol values is described in details elsewhere.^24^ The University College London ethics committee reviewed and approved the study. Written informed consent was obtained from all participants at each study phase.

### Statistical analyses

To describe secular trends, 10th, 50th and 90th percentiles of risk factors were calculated for the 57–61 age group at each of the last four phases (this age group was not represented in phase 1 when the participants were at 35–55 years of age). This resulted in an 18-year long study period for this sequential cross-sectional analysis. We investigated whether trends were similar across the full range of the distribution of each cardiovascular risk factor. A linear trend was estimated with quantile regression using calendar year as the explanatory variable. Non-parametric smooth kernel distributions were fitted to get an overall picture of how the characteristics (location and shape) changed from phase to phase.

Quadratic age-related risk factor trajectories for the mean were assessed with mixed-effects models with random intercepts. We added year of birth and its interaction terms with age and age-squared to the models to analyse secular trends. To test the robustness of our results against selective loss of follow-up and healthy survival, we fitted models to a subgroup who participated up to phase 9 (not necessarily in each phase) in a sensitivity analysis. Statistical analysis was performed using Mathematica 9 (Wolfram Research) and R version 2.15.1 (lme4, quantreg and Epi packages).

## Results

### Cohort characteristics

Baseline characteristics are shown in Table 1. Women were more likely to be current smokers than men. Almost half of the women had the lowest employment grade, whereas this was the case only in 1 in 10 men. The largest sex differences in cardiovascular risk factors were in WC and HDL.

**Table 1.** Baseline characteristics (1985–88). Values are medians (Q1; Q3) or percentages (%)

Characteristic	Men (*N* = 6895)	Women (*N* = 3413)
Age (year)	43 (39; 49)	45 (40; 51)
Whites (%)	91.5	84.2
Smoking (%)		
Never	47.3	52.9
Ex	36.1	23.3
Current	15.8	23.3
Missing	0.8	0.5
Grade level (%)		
Administrative	38.4	11.2
Prof/executive	52.3	39.1
Clerical/support	9.3	49.7
BMI (kg/m^2^)	24.3 (22.6; 26.2)	24.0 (21.9; 26.7)
WC (cm)^a^	88.9 (83.0; 94.7)	76.5 (69.5; 85.4)
SBP (mmHg)	123 (115; 133)	118 (109; 130)
DBP (mmHg)	77 (71; 84)	75 (68; 81)
TC (mmol/l)	5.9 (5.2; 6.7)	5.8 (5.1; 6.6)
HDL (mmol/l)	1.3 (1.1; 1.6)	1.6 (1.4; 2.0)

^a^WC values are from phase 3, when first measured.

The number of participants at each phase is displayed in Table 2. Relatively fewer men (31.0%) than women (41.3%) were lost (caused by either death or non-response/withdrawal) from the study until phase 9. Attrition was largest between phase 1 and 3 (12.8%). The average number of visits per participant was 4.

**Table 2.** Summary of participation status at each study phase. Number of participants and cumulative number of deaths and non-responses/withdrawals are reported

	**Status**	**Phase 1 1985–88**	**Phase 3 1991–94**	**Phase 5 1997–99**	**Phase 7 2002–04**	**Phase 9 2007–09**
Men	Participated	6895	6057	5473	4893	4759
	Died		81	204	389	621
	Non-response / withdrawal		757	1218	1613	1515^a^
Women	Participated	3413	2758	2397	2074	2002
	Died		44	102	195	333
	Non-response / withdrawal		611	914	1144	1078^a^

^a^Cumulative numbers may decrease, because we had information about the death of participants, even if they did not respond in a previous phase.

### Sequential cross-sectional analyses (age group: 57–61 years)

**Figure 1 A,B.**
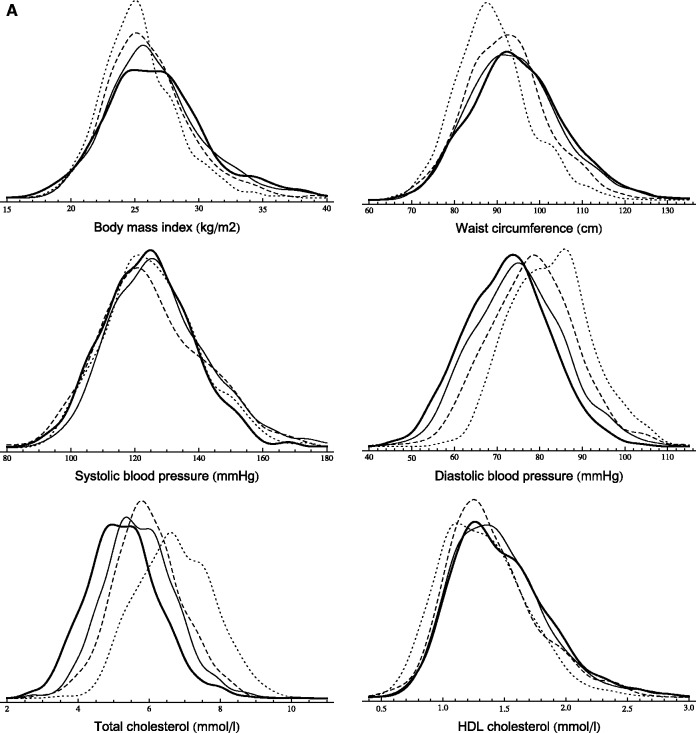

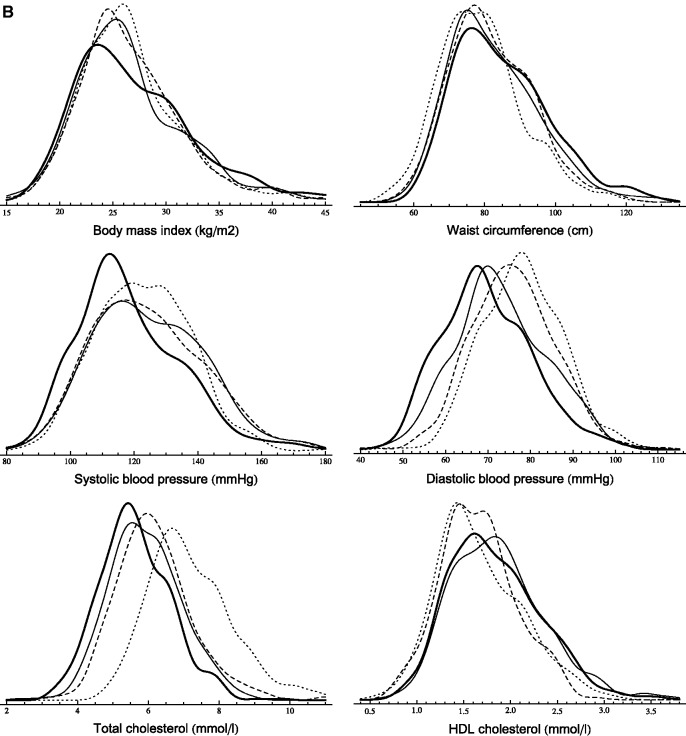
Sequential cross-sectional analysis (age group: 57–61 years). Smooth kernel distributions of cardiovascular risk factors (probability density functions are displayed) in men (A) and women (B) (dotted line: phase 3, dashed line: phase 5, solid line: phase 7, thick line: phase 9).

Table 3 displays cardiovascular risk factor percentiles (10th, 50th, 90th) in the 57–61 age group at study phases 3, 5, 7 and 9. Smooth kernel distributions are shown in Figure 1.

**Table 3A, B.** Sequential cross-sectional analysis (age group: 57–61 years). Secular trends of cardiovascular risk factors in men (A) and women (B). Linear trends were assessed with quantile regression models

**A**						
**Men**	**Percentile**	**Phase 3 1991–94 *N* = 911**	**Phase 5 1997–99 *N* = 903**	**Phase 7 2002–04 *N* = 1222**	**Phase 9 2007–09 *N* = 1391**	**Linear trend (per year)**
						**β (95% CI)**
BMI (kg/m^2^)	10th	21.9	22.2	22.4	21.9	0.004 (−0.014; 0.027)
	50th	25.1	25.6	26.1	26.4	0.080 (0.065; 0.096)***
	90th	29.1	30.4	31.8	31.9	0.192 (0.145; 0.234)***
WC (cm)	10th	78.4	80.3	81.2	80.6	0.152 (0.100; 0.210)***
	50th	88.2	91.9	93.6	94.2	0.360 (0.318; 0.409)***
	90th	101.4	105.2	108.4	109.6	0.508 (0.433; 0.631)***
SBP (mmHg)	10th	107	106	109	107	0.000 (−0.086; 0.092)
	50th	125	123	126	124	0.000 (−0.031; 0.083)
	90th	145	148	150	143	−0.181 (−0.313; −0.030)*
DBP (mmHg)	10th	71	65	61	60	−0.654 (−0.714; −0.603)***
	50th	82	78	75	73	−0.594 (−0.653; −0.517)***
	90th	95	91	89	85	−0.577 (−0.650; −0.490)***
TC (mmol/l)	10th	5.3	4.8	4.5	4.0	−0.079 (−0.085; −0.074)***
	50th	6.7	5.9	5.7	5.2	−0.083 (−0.088; −0.077)***
	90th	8.1	7.3	7.0	6.6	−0.086 (−0.093; −0.079)***
HDL (mmol/l)	10th	0.9	1.0	1.0	1.0	0.005 (0.003; 0.008)***
	50th	1.3	1.3	1.4	1.4	0.008 (0.004; 0.010)***
	90th	1.8	1.9	1.9	2.0	0.011 (0.009; 0.013)***

**B**						
**Women**	**Percentile**	**Phase 3 1991-1994 *N* = 520**	**Phase 5 1997-1999 *N* = 383**	**Phase 7 2002-2004 *N* = 492**	**Phase 9 2007-2009 *N* = 514**	**Linear trend (per year)**
						**β (95% CI)**
BMI (kg/m^2^)	10th	21.3	21.2	21.2	21.1	−0.007 (−0.043; 0.021)
	50th	26.0	25.8	25.8	26.1	0.000 (−0.023; 0.025)
	90th	32.8	32.5	33.8	34.8	0.120 (0.063; 0.205)**
WC (cm)	10th	64.9	68.0	68.1	69.9	0.289 (0.190; 0.351)***
	50th	78.4	80.0	81.0	83.0	0.276 (0.173; 0.391)***
	90th	96.0	96.3	100.2	104.0	0.482 (0.354; 0.657)***
SBP (mmHg)	10th	104	103	104	99	−0.277(−0.406; −0.091)**
	50th	122	123	123	116	−0.350 (−0.516; −0.202)***
	90th	141	148	147	141	0.094 (−0.141; 0.278)
DBP (mmHg)	10th	66	64	60	56	−0.634 (−0.707; −0.524)***
	50th	78	75	72	69	−0.558 (−0.647; −0.491)***
	90th	90	89	88	84	−0.359 (−0.464; −0.201)***
TC (mmol/l)	10th	5.8	4.9	4.7	4.4	−0.084 (−0.093; −0.071)***
	50th	7.0	6.1	5.9	5.5	−0.084 (−0.093; −0.078)***
	90th	8.8	7.6	7.3	6.9	−0.115 (−0.122; −0.098)***
HDL (mmol/l)	10th	1.2	1.2	1.3	1.3	0.009 (0.007; 0.010)***
	50th	1.6	1.6	1.8	1.8	0.012 (0.010; 0.019)***
	90th	2.3	2.2	2.5	2.5	0.015 (0.011; 0.023)***

*N* is the number of participants (aged 57–61 years) in each phase. Missing measures per variable do not exceed 3%, except phase 5 (BMI: 13%, WC: 20% and HDL: 13%), when timing and organization issues took place at the screening (so we can assume that values are missing at random).

*P < 0.05; **P < 0.01; ***P < 0.001.

The BMI distribution of men not only shifted to the right (except for extremely low percentiles) but also became more fat-tailed on the right. The increment was larger in higher percentiles (the 90th percentile increased by 2.8 kg/m^2^ between phases 3 and 9), which led to a higher degree of skewness. The change in women showed a slightly different pattern. A larger part of the distribution’s left end (below 23 kg/m^2^) remained unchanged, but a fat-tail grew on the right end (the 90th percentile increased by 2 kg/m^2^). Contrarily to BMI, WC increased also in the 10th percentile, but the changes were the largest in the right end of the distribution with an ∼8-cm increment between 1991 and 2009 in both men and women.

SBP measures slightly increased until phase 7, but dropped markedly at the last phase. The entire DBP distribution shifted to the left by 10 mmHg in men. In women, the decline was larger in the 10th percentile than in the 90th (10 mmHg vs 6 mmHg).

There was a marked distribution shift to the left in TC. The median decreased by ∼22% in both men and women. This 1.5-mmol/l decline characterized the entire distribution, although the 90th percentile decreased even more (1.9 mmol/l) in women. The changes in the HDL distribution were modest compared with our observations in TC, although all calculated percentiles increased by 0.1–0.2 mmol/l.

Changes in smoking habits and medication usage are shown in Table 4. The percentage of current smokers remained constant at ∼8% in men, but decreased markedly in women. Usage of lipid-lowering medication increased drastically in both men and women. Whereas at phase 3 only 1–2% took medication, at phase 9 every fourth man and fifth woman did so. Around 25% of participants were on antihypertensive medication at phase 9.

**Table 4A, B.** Sequential cross-sectional analysis (age group: 57–61 years). Secular trends of lipid-lowering and antihypertensive medication and smoking habits in men (A) and women (B)

A				
Men	Phase 3 1991–94	Phase 5 1997–99	Phase 7 2002–04	Phase 9 2007–09
Current smoker (%)	8.5	8.8	8.7	7.9
Lipid treatment (%)	1.4	4.1	10.6	24.0
Antihypertensive treatment (%)	12.4	15.2	21.9	28.0

B				
Women	Phase 3 1991–94	Phase 5 1997–99	Phase 7 2002–04	Phase 9 2007–09
Current smoker (%)	15.9	10.6	11.3	4.7
Lipid treatment (%)	2.0	4.1	7.9	19.9
Antihypertensive treatment (%)	16.9	20.9	20.9	24.8

### Longitudinal analyses

**Figure 2A, B.**
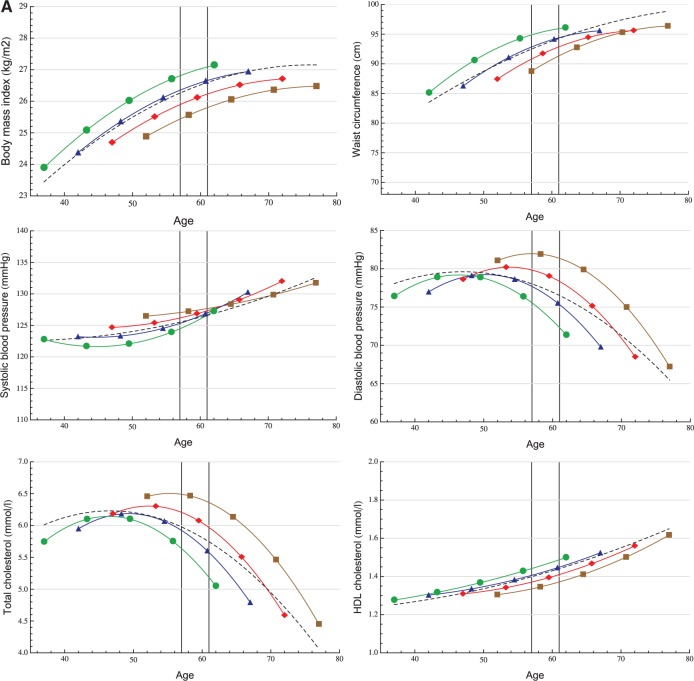

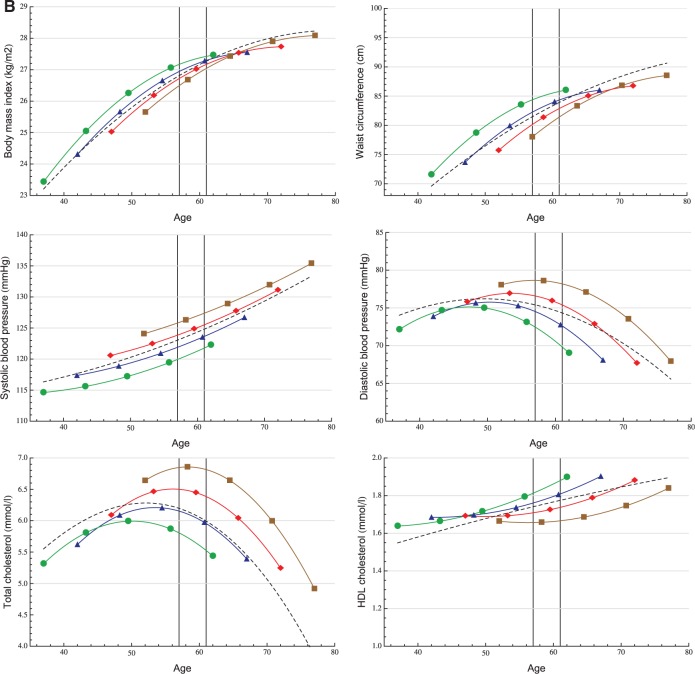
Longitudinal trajectory analysis. Age-related trajectories (in years) of cardiovascular risk factors in men (A) and women (B) with adjustment for four different birth cohorts: 1933 (▪), 1938 (♦), 1943 (▴), 1948 (•) and unadjusted (– – –).

Age-related mean risk factor trajectories for the entire study population are displayed in Figure 2. The age range of 57–61 years is highlighted to visualize the subset of participants used for the quantile regression analyses. Model coefficient estimates are displayed in eTable 1 (available as Supplementary data at *IJE* online).

Both BMI and WC increased faster in younger generations and were at higher levels than in older birth cohorts at any given age {e.g. a BMI difference at age 60 between those born in 1933 and 1948 of 1.3 kg/m^2^ [95% confidence interval (CI) 1.1; 1.5] and 0.5 kg/m^2^ [95% CI 0.1; 0.9] for men and women, respectively}. The secular trend in BMI was greater in men than in women, whereas women in all birth cohorts had steeper BMI and WC trajectories compared with men.

SBP increased faster with age in women than in men: 15.1 mmHg vs 8.9 mmHg, respectively, between ages 40 and 75 (12.9 % vs 7.2% relative difference). Younger generations, except men in late adulthood, had lower SBP levels. The mean DBP trajectory (unadjusted) increased until age 50, and then decreased markedly in both men and women. The peaks of the adjusted DBP trajectories occurred at earlier ages in younger generations, who also had generally lower DBP levels: at age 60, men born in 1948 had a 8.4-mmHg (95% CI 7.8; 9.0) lower DBP than men born in 1933, whereas for women this difference was 7.8 mmHg (95% CI 7.0; 8.7).

The unadjusted TC trajectory in men increased up to age 47, peaking at 6.2 mmol/l (95% CI 6.2; 6.3) before declining. For women the corresponding peak value was 6.3 mmol/l (95% CI 6.3; 6.4) at age 52. Men born in 1948 had 1.1-mmol/l (95% CI 1.0; 1.2) lower TC level at age 60, than men born in 1933, whereas for women the difference between these birth cohorts was 1.2 mmol/l (95% CI 1.1; 1.3). Women had higher HDL levels during the entire age range under examination. We also observed a modest positive trend and increment with ageing in both sexes.

In sensitivity analyses we fitted longitudinal models to a subsample of participants who attended up to the last phase, and we found associations very similar to those described above (data not shown).

## Discussion

We found that middle-aged British men and women experienced a marked change in cardiovascular risk over the past 25 years, which was partly attributable to ageing and partly due to secular trends. We report that at any given age younger generations were more obese, but had a more favourable lipid profile (i.e. lower total cholesterol levels and higher HDL cholesterol levels). Furthermore, the observed secular trends affected risk factor distributions. Whereas the increase in obesity led to more right-skewed distributions, the decrease in total cholesterol was characterized by a left-shift of the entire distribution.

### Obesity

We found evidence that changes in obesity were heterogeneous across the population between 1985 and 2009. Our findings show that BMI increased particularly in groups already affected by obesity, but also that although BMI did not change in leaner groups, these were still developing abdominal obesity. It is conceivable that as people become increasingly sedentary, leaner people lose muscle mass and simultaneously accumulate abdominal fat mass, leaving their BMI unchanged while their WC increases. Given the particular impact of central obesity on glucose metabolism^25^ and mortality^26^ independently of general obesity, our findings highlight the importance of assessing WC particularly among people with BMI levels in the normal or overweight range.^25^

The global obesity ‘epidemic’ has been documented in several countries, based on studies reporting mean BMI levels^17^ or the prevalence of overweight and obesity.^27^ The BMI distribution became more right-skewed between adolescence and early adulthood^28^ and in adulthood^20^^,^^21^ in US and Canadian populations.^29^ In addition, a Chinese population-based study showed that the WC distribution shifted to the right.^22^

As the marked increase in obesity in the UK seems to lag behind the developments in North America, where the most recent results show that the epidemic is levelling off,^30^ it is conceivable that the right-skewing of the BMI distribution we observed is a feature of the early dynamic phase of the obesity epidemic, and that there is a point at which the divergence of percentiles stabilizes.

Our analysis of BMI and WC trajectories showed that in the past 25 years in the UK, each successive generation has reached a set level of both obesity measures at an earlier age. Participants born as little as 15 years apart (1948 compared with 1933) reached overweight at a 10-year earlier age for men and a 6-year earlier age for women. It is clear that the 1933 generation in the UK have had a very different early life experience, spending their youth in the austerity of the 1930s and the war period, whereas the 1948 generation spent these same years in the years of post-war reconstruction, increasing welfare and food security. However, evidence from younger cohorts suggests that this development has not yet ceased and that even current teenagers are taller and more obese than teenagers a decade ago.^31^

A recent analysis from the CARDIA study^32^ showed that not only the attained level, but also the duration of overall and abdominal obesity, were associated with coronary artery calcification indicating that subsequent birth cohorts may be at elevated cardiovascular risk at a younger age.

We showed that increasing abdominal obesity affects both sexes similarly, and confirmed previous findings that BMI levels in men are catching up with those in women.^12^^,^^13^

A previous study on BMI trajectories reported that age-related increases in BMI are underestimated using solely cross-sectional data.^14^ In addition to this, we argue that using longitudinal analyses without appropriate adjustment for secular trends might also lead to biased estimates as a consequence of large differences between different birth cohorts.

### Blood pressure

Our results are in line with the current understanding that SBP rises from mid to late adulthood, whereas DBP peaks around age 50 years and then starts to decline as a result of increasing aortic and small vessel stiffness.^33^^,^^34^ These previous reports were also based on longitudinal studies, but did not consider secular trends. By explicitly modelling trajectories for different birth cohorts, we were able to show that DBP levels are generally lower for younger cohorts. In contrast, the dynamics of SBP trend were not consistent. Although the results of mean trajectory analyses showed a declining trend in women’s SBP (see Figure 2), the quantile regression models suggest that this was mainly driven by changes between the last two phases. Both our sequential cross-sectional and trajectory analyses confirm the magnitude of DBP drop during the past 25 years and also with ageing. This drop appears to be starting earlier in life in younger generations. As DBP decreases faster across generations compared with SBP, a widening gap between SBP and DBP develops leading to a larger and faster increase of pulse pressure with age in younger compared with older cohorts, especially among men. As pulse pressure is an independent determinant of coronary heart disease (CHD) risk^35^and DBP has been shown to have a negative association with CHD risk after the age of 60 years,^36^ caution is required when interpreting secular trends in blood pressure.

### Cholesterol

Previous studies have reported that TC levels are declining in high-income countries^9^ including the USA^4^ and England,^37^ An earlier report from the Whitehall II study,^24^ in line with reports from other developed countries,^38^ found that the decline in LDL cholesterol could not be fully attributed to increasing use of lipid-lowering medication and that improvements in diet play a significant role.

We confirm these earlier observations by reporting a progressive left-shift of the entire TC distribution. Although the observed decline may be at least partially due to the wider use of statins in the past decades, particularly in the elderly, this could not explain a decreasing trend in the lower extremes of the distribution. This probably indicates that secular trends in other factors (e.g. diet, lifestyle) may have played an important role. We also have to note that medication use increased both with age and calendar year, leading to younger generations being more likely to get lipid-lowering medication earlier in their life.

The effect of secular trends on cholesterol levels was so large in the Whitehall II study that the unadjusted age-related trajectory is clearly biased and underestimates TC levels in late adulthood. This implies that an individual’s multiple measurements over a longer period should not be compared with population average trajectories without adjustment for the birth cohort effect. The magnitude of secular trends in cholesterol levels over the past three decades also makes this a particularly challenging period for the study of the isolated effects of ageing on cholesterol levels.

We observed a positive HDL cholesterol trend, but it was very modest compared with changes seen in TC levels. In particular, it seems that younger generations of women have more favourable HDL levels throughout later middle age, reflected by higher and steeper trajectories compared with earlier birth cohorts.

### Strengths

The key strength of our analysis was that we could study the effect of secular trends and ageing on several cardiovascular risk factors simultaneously in a relatively large population-scale sample with up to five repeated measurements over a 25-year period. Contrary to the common approach of reporting average values and standard deviations, we applied quantile regression which is well suited to highlight changes in distribution characteristics.

### Limitations

Loss to follow-up is a critical question when analysing data from a longitudinal study with a long follow-up period. There is no test to determine whether data are missing at random (MAR) or missing not at random (MNAR); thus both cases should be considered. If the MAR assumption holds, likelihood-based methods give valid estimates. If the missingness pattern is MNAR, sensitivity analyses should be applied.^39^ In this study, such analyses replicated the main findings, suggesting that our results were robust (data not shown). Standardization of laboratory and other measurements is a recognized problem. The changes in blood pressure measurement device is an unlikely cause of the secular trend we showed, as AOD produces slightly higher values than MRZ.^40^ Although all labs participated in the National External Quality Assurance Scheme for TC and HDL, we cannot entirely exclude standardization issues. The 57–61 age group was arbitrarily chosen, so our conclusions about risk factor distribution characteristics are not general for adulthood. Sex differences could be biased by the uneven distribution of men and women across employment grades. Previous studies showed lower mortality rates^41^ and systolic blood pressure trajectories,^33^ but similar BMI trajectories^33^ in our occupational cohort compared with population-based cohorts. Although it is likely that other risk factor levels may also be slightly more favourable in the Whitehall II cohort, this does not imply that secular trends are also more favourable.

## Conclusion

We showed in an occupational cohort of British civil servants that the past three decades brought strong and heterogeneous secular trends in obesity, blood pressure and lipid levels. Decreasing CVD mortality rates in the UK suggest that negative trends in obesity are counterbalanced by favourable changes in blood pressure and cholesterol levels. The disparity of the trends indicates that the relative importance of these risk factors is likely to change over time. This observation has potential implications for the way we assess global CVD risk based on risk scores, highlighting the need to reassess the calibration of component weights on a regular basis. Our findings also suggest that more detailed models of risk factor progression may be needed to help track the development not only of average levels but also changing risk factor distributions in the population. Not accounting for secular trends may cause biased estimates in longitudinal analyses of age-related risk factor trajectories.

## Supplementary Data

Supplementary material is available at *IJE* online.

## Funding

The Whitehall II study is supported by grants from the Medical Research Council (K013351), British Heart Foundation (RG/13/2/30098), National Heart Lung and Blood Institute (RO1 HL036310) and National Institute of Aging (RO1AG13196 and RO1AG034454). The authors thank the staff and participants of the Whitehall II study for their important contributions. A.H. is supported by the TÁMOP 4.2.2.A-11/1/KONV-2012-0052 Program. A.G.T. is supported by the TÁMOP 4.2.4.A/1-11-1-2012-0001 National Excellence Program – research fellowship co-financed by the European Union and the European Social Fund.

**Conflicts of interest:** Steno Diabetes Center A/S receives part of its core funding from unrestricted grants from the Novo Foundation and Novo Nordisk A/S. D.V. is employed by Steno Diabetes Center A/S, a research hospital working in the Danish National Health Service and owned by Novo Nordisk A/S. D.V. and D.R.W. own shares in Novo Nordisk A/S.

## Supplementary Material

Supplementary Data
