# Supplementary material for: Effect of secular trends on age-related trajectories of cardiovascular risk factors: the Whitehall II longitudinal study 1985–2009
Source: Int J Epidemiol. 2014 Jan 24;43(3):866–77. doi: 10.1093/ije/dyt279 (PMC4052135; doi:10.1093/ije/dyt279)
Supplement: Supplementary Data [file supp_43_3_866__index.html]

Effect of secular trends on age-related trajectories of cardiovascular risk factors: the Whitehall II longitudinal study 1985–2009 — Supplementary Data 

# Effect of secular trends on age-related trajectories of cardiovascular risk factors: the Whitehall II longitudinal study 1985–2009

## Supplementary Data

files

**Files in this Data Supplement:**

- Supplementary Data - pdf file
