# Supplementary material for: Effect of secular trends on age-related trajectories of cardiovascular risk factors: the Whitehall II longitudinal study 1985–2009
Source: Int J Epidemiol. 2014 Jan 24;43(3):866–77. doi: 10.1093/ije/dyt279 (PMC4052135; doi:10.1093/ije/dyt279)
Supplement: Supplementary Data [file supp_dyt279_eTable1updated.pdf]

Electronic Supplementary Material

eTable1 A,B Estimated coefficients in age-related trajectories (mixed-effects models) of cardiovascular risk factors for men (A) and women (B). Age and year of birth (YOB) were centered around 60 years and 1940, respectively.

A

	Intercept	Age	Age^2	YOB	YOB x Age	YOB x Age^2
BMI (kg/m^2)	26.33***	0.0750***	-0.0028***	0.0875***	-0.0015***	-0.00005***
WC (cm)	93.05***	0.4286***	-0.0183***	0.3333***	-0.0261***	-0.00027***
SBP (mmHg)	126.8***	0.3567***	0.0113***	-0.0989***	0.0271***	0.00093***
DBP (mmHg)	77.70***	-0.5267***	-0.0345***	-0.5602***	-0.0433***	0.00032***
TC (mmol/l)	5.897***	-0.0790***	-0.0044***	-0.0730***	-0.0054***	-0.00001
HDL (mmol/l)	1.415***	0.0106**	0.0002***	0.0076***	0.0001	-0.00001***

B

	Intercept	Age	Age^2	YOB	YOB x Age	YOB x Age^2
BMI (kg/m^2)	27.13***	0.0940***	-0.0043***	0.0307*	-0.0051***	-0.00010***
WC (cm)	82.91***	0.5731***	-0.0230***	0.3263***	-0.0336***	-0.00040**
SBP (mmHg)	124.3***	0.4379***	0.0066**	-0.3788***	0.0047	0.00018
DBP (mmHg)	74.74***	-0.4248***	-0.0272***	-0.5205***	-0.0366***	-0.00013
TC (mmol/l)	6.265***	-0.0466***	-0.0048***	-0.0821***	-0.0039***	0.00011***
HDL (mmol/l)	1.756***	0.0099***	0.0004***	0.0133***	0.0009***	-0.00001

*p<0.05, **p<0.01, ***p<0.001

BMI, Body mass index; WC, Waist circumference; SBP, Systolic blood pressure; DBP, Diastolic blood pressure; TC, Total cholesterol; HDL, HDL cholesterol.
